# Influence of the seed of measurement on the work extracted in a quantum Szilard engine

**DOI:** 10.1016/j.isci.2023.108563

**Published:** 2023-11-24

**Authors:** Marina Cuzminschi, Alexei Zubarev, Stefan-Marian Iordache, Aurelian Isar

**Affiliations:** 1 Department of Theoretical Physics, “Horia Hulubei” National Institute for Physics and Nuclear Engineering, 07125 Magurele, Ilfov, Romania; 2 Faculty of Physics, University of Bucharest, 077125 Magurele, Ilfov, Romania; 3 Plasma Physics and Nuclear Fusion Laboratory, National Institute for Laser, Plasma and Radiation Physics, 077125 Magurele, Ilfov, Romania; 4 Extreme Light Infrastructure, National Institute for Physics and Nuclear Engineering, 07125 Magurele, Ilfov, Romania; 5 Optospintronics Department, National Institute for Research and Development for Optoelectronics—INOE 2000, 077125 Magurele, Ilfov, Romania

**Keywords:** Physics, Quantum theory, Quantum measurement

## Abstract

We investigate the influence of the seed of measurement on the performance of a Szilard engine based on a two-mode Gaussian state evolving in a noisy channel. Quantum work is extracted by performing a positive operator-valued measurement (POVM) on one of the two modes, after which this mode reaches equilibrium with the environment. As the seed of measurement, we use a single-mode squeezed thermal state. We employ the Markovian Kossakowski-Lindblad master equation to determine the evolution in time of the considered open system and the quantum work is defined based on the Rényi entropy of order 2. We show that the extracted quantum work and information-work efficiency strongly depend on the characteristic parameters of the system (frequency, average thermal photons number, and squeezing), the noisy channel (temperature and squeezing of the bath), and the seed of measurement (average thermal photons number and strength of the measurement).

## Highlights

• A bipartite Gaussian state is used as working medium for a Szilard engine
• A single-mode squeezed thermal state is used as the seed of measurement
• Expression of extracted work is deduced for a standard form covariance matrix
• Dynamics of extracted work and work-information efficiency is investigated

## Graphical abstract

**Figure:**
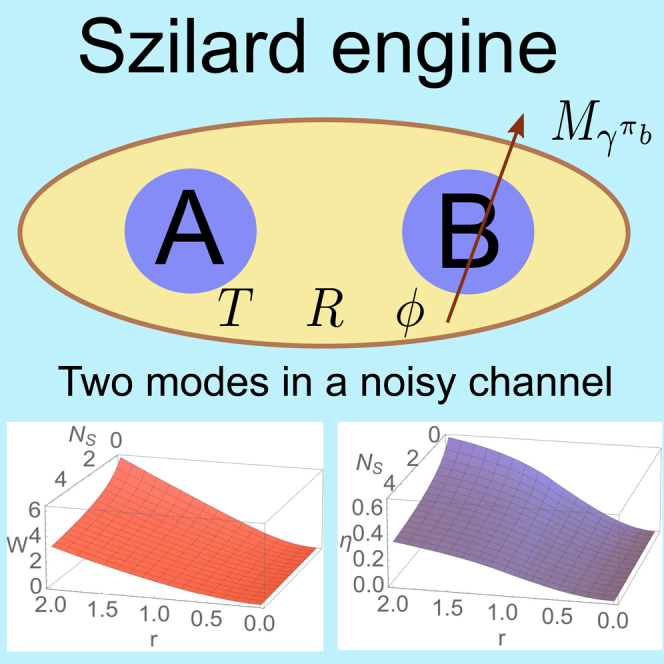

## Introduction

The single-molecule Szilard engine was proposed in 1929 by Leo Szilard as a hypothetical device for conversion of internal energy of a reservoir into work.^1^ Its design is the following: a single molecule is confined in a box and it is weakly interacting with a thermal reservoir of temperature *T*.^1^ A mobile membrane is inserted in the middle of the box. Following this step, a measurement is performed to check in which part of the box the molecule is found. Making use of the measurement result, by attaching a load to the membrane, one can extract work by an isothermal expansion. In this process, an amount of work up to $k_{B}T\;\ln\;2$ can be extracted, where $k_{B}$ is the Boltzmann constant.^2^^,^^3^^,^^4^ After the work extraction, the mobile membrane is removed, the initial state of the engine is restored, and the working cycle is completed.

The paradox of Szilard engine functionality is the following: the work is extracted from the system even though the free energy difference between the initial and final state is zero. This signals the violation of the second law of thermodynamics and suggests taking into account energy loss during the measurement process.^5^^,^^6^^,^^7^^,^^8^ According to the Landauer principle, any erasure of information about the measured system requires an energy of at least $k_{B}T\;\ln\;2$ per bit. The Landauer principle has been experimentally validated for a single colloidal particle.^8^ In this way, the agreement of a classical Szilard engine functionality with the second law of thermodynamics is reinforced. As a result, we can proclaim that a classical Szilard engine cannot produce work or be used as an energy source. All the extracted work left after we measure the position of the molecule will dissipate as heat after erasing the memory used for the storage of measurement results.^9^ Therefore, special attention should be paid to the quantum Szilard engine study.^10^

A few experimental realizations of the Szilard engine have been achieved.^11^^,^^12^^,^^13^ In ref. 2 it was shown that by using a bit of information the extracted work is $k_{B}T\;\ln\;2$. The experimental setup consists of a single electron in a box composed of two small metallic islands connected by a tunnel junction. The box electrodes contain several electrons, and the position of one extra electron determines the charge configuration of the box. The information is encoded in the position of the extra electron. Moreover, using the same experimental setup of a single electron in a box, the role of the mutual information in the fluctuation theorem has been experimentally validated.^14^

It has been demonstrated^15^ that more work can be extracted out of a heat bath via entangled systems than via classically correlated systems. Experimental study of bipartite and multipartite entangled states with multi-photon optical interferometers confirms the quantum correlations advantage.^16^

Other theoretical investigations regarding Szilard engine^17^^,^^18^^,^^19^ and Stirling engine^20^^,^^21^ have been performed in recent years. In study,^18^ a quantum Szilard engine with particle under the influence of the fractional power-law potential has been investigated. It has been demonstrated that this engine works like a Stirling-like cycle. Energy eigenvalues and canonical partition functions have been derived for both degenerate and non-degenerate cases. Stirling engine represents a modified version of quantum Szilard engine, includes one or more particles, and can be put in functionality using quantum features, such as energy degeneracy. Quantum Stirling cycle that operates using quantized energy levels of a potential well has been investigated in study.^20^ The amount of extractable work from engines based on distinguishable particles, fermions, and bosons is calculated. The obtained engines efficiencies are comparable to the corresponding Carnot efficiencies in the low temperature limit. In Ref. ^21^ Stirling-like cycle with a single particle put under the influence of infinite potential well has been explored. Work and engine efficiency can be greatly influenced by the length of the potential well and fractional exponent of the engine.

The work extraction using bipartite correlated Gaussian states in the quantum Szilard engine as a working medium has been discussed in Refs. ^22^^,^^23^^,^^24^ It was shown that it is possible to use the extracted work for entanglement and steering detection. In Ref. ^22^ we assumed the following working model for Gaussian state-based quantum Szilard engine: Alice and Bob share an initially entangled bimodal Gaussian state in a noisy channel and the Bob mode is measured. Due to backreaction, Alice mode state changes. After that, Alice mode is left to expand isothermally and during this process the quantum work can be extracted.

Gaussian states are attractive for designing quantum devices due to the fact that they can be prepared and manipulated easily in the laboratory,^25^ by using laser sources.^26^ For comparison, obtaining single-photon states and two-photon entangled states can be technically complicated,^25^ and manipulating qubits based on solid state devices can require temperatures as low as a few mK.^2^^,^^27^

There are two main types of Gaussian measurements: projective (von Neumann) measurement and positive operator-valued measurement (POVM), which is a more general class compared to projective measurements.^26^ POVM form includes the thermal noise influence upon the measured system, which is unavoidable in laboratory experiments or devices.^28^

In Refs. ^22^^,^^23^^,^^24^ it was shown that the quantity of extracted work and its efficiency significantly depend on the type of measurement used in the Szilard engine. In particular, the homodyne and heterodyne measurements were compared. In the present article we consider a unimodal squeezed thermal state as the seed of measurement for the POVM. We describe the influence of the characteristic parameters of the system (frequency, average number of thermal photons and squeezing), of the noisy channel (temperature and squeezing of the bath), and of the seed of measurement (average number of thermal photons and strength of the measurement) on the extracted work and efficiency of the Szilard engine.

The article is organized in the following way. In section “extracted work and information-work efficiency” the role of the seed of measurement on the extracted quantum work and work-information efficiency is analyzed. In section “Szilard engine performance” the behavior of the extracted work and information-work efficiency as functions of the parameters characterizing the two-mode system, the noisy channel, and the seed of measurement is presented. Finally, the obtained results are summarized in “conclusions”.

### Extracted work and information-work efficiency

We consider a bipartite quantum system *AB* consisting of two modes $\hat{a}$ and $\hat{b}$ evolving in a Gaussian noisy channel, and characterized by the covariance matrix in the standard form^26^^,^^29^^,^^30^:

(Equation 1) $$\sigma \left(t\right)=\left(\begin{array}{cc} \sigma_{a}\left(t\right) & \sigma_{ab}\left(t\right)\\ \sigma_{ab}^{T}\left(t\right) & \sigma_{b}\left(t\right) \end{array}\right)\equiv \left(\begin{array}{cccc} a\left(t\right) & 0 & c\left(t\right) & 0\\ 0 & a\left(t\right) & 0 & d\left(t\right)\\ c\left(t\right) & 0 & b\left(t\right) & 0\\ 0 & d\left(t\right) & 0 & b\left(t\right) \end{array}\right)\text{,}$$

where $\sigma_{a}$ and $\sigma_{b}$ represent the covariance matrices of the two modes and $\sigma_{ab}$ contains the correlations between them.

Terms a(t),b(t)≥1, $\left[\left(a^{2}\left(t\right)-1\right)\left(b^{2}\left(t\right)-1\right)-2c\left(t\right)d\left(t\right)-a\left(t\right)b\left(t\right)c^{2}\left(t\right)+d^{2}\left(t\right)\left(c^{2}\left(t\right)-a\left(t\right)b\left(t\right)\right)\right]\geq 0$ and we can also set the condition c(t)≥|d(t)|. These criteria guarantee that the bona fide conditions are ensured.^26^ Any Gaussian state using local unitary (symplectic) operations can be brought into a standard form.

We can denote:

(Equation 2) $$\begin{array}{cc} & I_{1}=\det\;\sigma_{a}\left(t\right)\text{,}\\ & I_{2}=\det\;\sigma_{b}\left(t\right)\text{,}\\ & I_{3}=\det\;\sigma_{ab}\left(t\right)\text{,}\\ & I_{4}=\det\;\sigma \left(t\right)\text{.} \end{array}$$

After that, using the relations

(Equation 3) $$I_{1}=a^{2}\left(t\right),I_{2}=b^{2}\left(t\right),I_{3}=c\left(t\right)d\left(t\right),I_{4}=\left(a\left(t\right)b\left(t\right)-c\left(t\right)\right)\left(a\left(t\right)b\left(t\right)-d\left(t\right)\right)\text{,}$$

to make the transition to the standard form of any bimodal covariance matrix.

**Figure 1:**
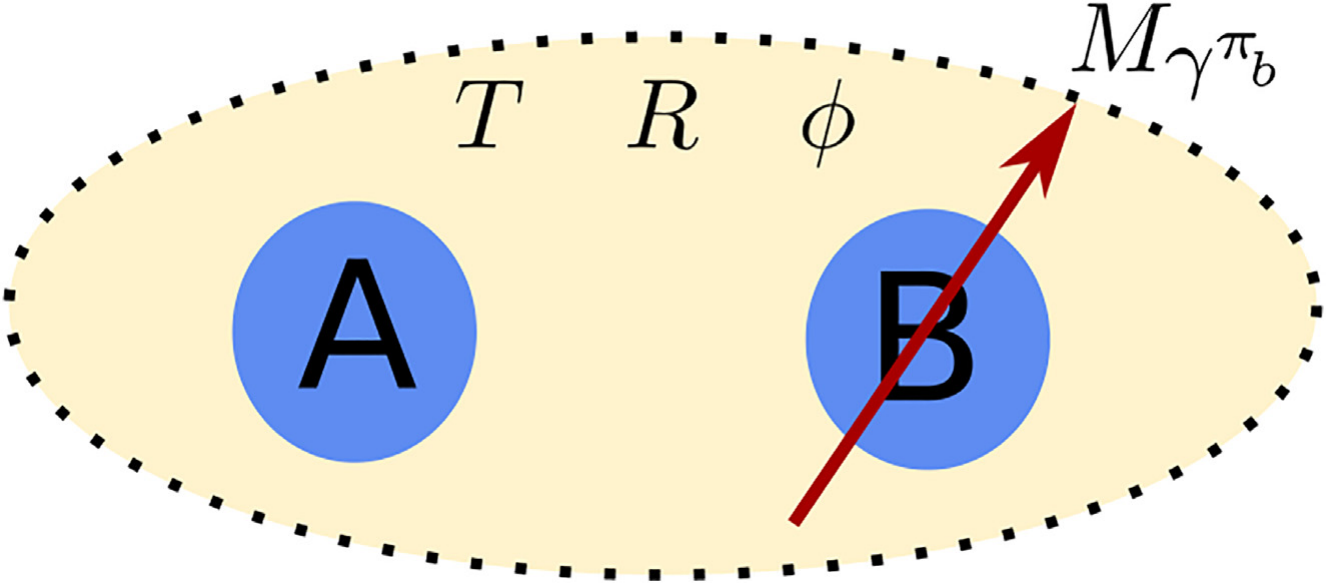
The schematic diagram of the considered Szilard engine The working medium is in a two-mode Gaussian state. One mode belongs to party A and the other one to party B. Both of them evolve in a Gaussian noisy channel characterized by temperature *T*, squeezing parameter *R*, and phase φ. Party B performs on his mode a POVM characterized by the seed of measurement $\gamma^{\pi_{b}}$.

The schematic diagram of the considered Szilard engine is presented in Figure 1.

Party B performs on his mode a Gaussian POVM of the form $\pi_{b}\left(X\right)=\pi^{-1}D_{b}\left(X\right)\rho^{\pi_{b}}D_{b}^{†}\left(X\right)$,^23^ where $D_{b}\left(X\right)=\exp\;\left(X{\hat{b}}^{†}-X^{*}\hat{b}\right)$ is the displacement Weyl operator, and $\rho^{\pi_{b}}$ is the density matrix of the seed of the measurement.^26^^,^^31^ The seed of measurement is a (generally mixed) single-mode Gaussian state with covariance matrix $\gamma^{\pi_{b}}$, with the first moments of the quadratures taken zero. In the previous works, as the seed of measurement for the studied Szilard engines was taken a squeezed coherent state, described by its corresponding covariance matrix.^22^^,^^23^^,^^24^^,^^32^ In the following, we will consider the more realistic case of a squeezed thermal state, with the density operator^28^:

(Equation 4) $$\rho^{\pi_{b}}=S\left(\xi \right)\nu S^{†}\left(\xi \right)\text{,}$$

where S(ξ) is the squeezing operator, ξ=ϱexpiθ, *ϱ* is the squeezing parameter of the seed of measurement, θ the phase, and ν is a thermal state with the average photon number $N_{S}$. Therefore, the covariance matrix of the single-mode squeezed thermal state has the following matrix elements^33^:

(Equation 5) $$\begin{array}{cc} & \gamma_{11}^{\pi_{b}}=\frac{2N_{S}+1}{2}\left(\cosh\;2\;\varrho +\sinh\;2\;\varrho \;\cos\;\theta \right)\text{,}\\ & \gamma_{22}^{\pi_{b}}=\frac{2N_{S}+1}{2}\left(\cosh\;2\;\varrho -\sinh\;2\;\varrho \;\cos\;\theta \right)\text{,}\\ & \gamma_{12}^{\pi_{b}}=\gamma_{21}^{\pi_{b}}=-\frac{2N_{S}+1}{2}\sinh\;2\;\varrho \;\sin\;\theta \text{.} \end{array}$$

The conditional state of party A does not depend on the measurement result, i.e., $\sigma_{a|X}^{\pi_{b}}\equiv \sigma_{a}^{\pi_{b}}$ and it is given by the Schur complement^23^^,^^26^^,^^31^^,^^34^:

(Equation 6) $$\sigma_{a}^{\pi_{b}}=\sigma_{a}-\sigma_{ab}{\left(\sigma_{b}+\gamma^{\pi_{b}}\right)}^{-1}\sigma_{ab}^{T}\text{.}$$

After the measurement, the party A reaches this new state, out of equilibrium, and it comes back into an equilibrium state after interacting with the thermal bath.^23^ We consider that the equilibrium state has the same energy as the initial state of the mode $\hat{a}$, so that $\sigma_{a}^{\text{eq}}=\sigma_{a}$. In this way, the extracted work is the result of the measurement backreaction^23^ and the work can be extracted in the process of reaching the equilibrium of the party A.

Since the state is independent of the measurement result, its average entropy is $\int dXp_{X}S\left(\sigma_{a|X}^{\pi_{b}}\right)=S\left(\sigma_{a}^{\pi_{b}}\right)$. Then the extractable work can be defined as^23^:

(Equation 7) $$W=k_{B}T\left[S\left(\sigma_{a}\right)-S\left(\sigma_{a}^{\pi_{b}}\right)\right]\text{.}$$

To quantify the entropy of the conditional state (6) that party A reaches due to the backreaction of the measurement of the party B, we use the Rényi entropy of order 2, $S\left(\rho \right)=-\text{lnTr}\left(\rho^{2}\right)$,^35^ that in the case of Gaussian states is a fully legitimate entropy functional given by

(Equation 8) $$S\left(\sigma_{ab}\right)=\frac{1}{2}\ln\left(\det\;\sigma_{ab}\right)\text{.}$$

Then the expression of the work (7) becomes^23^^,^^24^

(Equation 9) $$W=\frac{k_{B}T}{2}\ln\left(\frac{\det\;\sigma_{a}}{\det\;\sigma_{a}^{\pi_{b}}}\right)\text{.}$$

We underline that a nonzero *W* corresponds to the existence of classical correlations between the two subsystems A and B.^23^

As the initial state of the system AB we choose a general Gaussian state given by:

(Equation 10) $$\sigma_{init}=\left(\begin{array}{cccc} a_{I} & 0 & c_{I} & 0\\ 0 & a_{\text{II}} & 0 & c_{\text{II}}\\ c_{I} & 0 & b_{I} & 0\\ 0 & c_{\text{II}} & 0 & b_{\text{II}} \end{array}\right)\text{,}$$

with the matrix elements:

(Equation 11) $$a_{I}=\frac{1}{\omega_{1}}\left(n_{1}\;\cosh^{2}\;r+n_{2}\;\sinh^{2}\;r+\frac{1}{2}\cosh\;2\;r\right)\text{,}$$

(Equation 12) $$a_{\text{II}}=\omega_{1}\left(n_{1}\;\cosh^{2}\;r+n_{2}\;\sinh^{2}\;r+\frac{1}{2}\cosh\;2\;r\right)\text{,}$$

(Equation 13) $$b_{I}=\frac{1}{\omega_{2}}\left(n_{1}\;\sinh^{2}\;r+n_{2}\;\cosh^{2}\;r+\frac{1}{2}\cosh\;2\;r\right)\text{,}$$

(Equation 14) $$b_{\text{II}}=\omega_{2}\left(n_{1}\;\sinh^{2}\;r+n_{2}\;\cosh^{2}\;r+\frac{1}{2}\cosh\;2\;r\right)\text{,}$$

(Equation 15) $$c_{I}=\frac{1}{2\sqrt{\omega_{1}\omega_{2}}}\left(n_{1}+n_{2}+1\right)\sinh\;2\;r\text{,}$$

(Equation 16) $$c_{\text{II}}=-\frac{\sqrt{\omega_{1}\omega_{2}}}{2}\left(n_{1}+n_{2}+1\right)\sinh\;2\;r\text{,}$$

where $\omega_{1}$ and $\omega_{2}$ are the frequencies of the two bosonic modes $\hat{a}$ and $\hat{b}$, $n_{1}$ and $n_{2}$ are their average thermal photon numbers, and *r* denotes the squeezing parameter. In the case of $\omega_{1}=\omega_{2}=1$ this state reduces to squeezed thermal state. And if we consider $n_{1}=n_{2}=1$ this state becomes a squeezed vacuum state.

The information-work efficiency of a Szilard engine^36^ is determined as the ratio of the extracted work to the erasure work:

(Equation 17) $$\eta =\frac{W}{W_{eras}}\text{.}$$

The erasure work $W_{eras}$ is proportional to the information stored in the whole system (composed of the two subsystems A and B):

(Equation 18) $$W_{eras}=k_{B}T\;\ln\;2^{H\left(P\right)}\text{,}$$

where $H\left(P\right)=-\sum_{i=1}^{n}P_{i}\;\ln\;P_{i}$ is the Shannon entropy and associated with the probability $P_{i}$ distribution. To calculate the information-work efficiency we use the von Neumann entropy as the counterpart of the Shannon entropy to express the erasure work.^37^ In quantum mechanics the probability distributions are retrieved by the density operators ρ, and the von Neumann entropy is given by:

(Equation 19) S(ρ)=−Tr(ρlnρ).

For a two-dimensional Gaussian state $\rho_{G}$ the von Neumann entropy is given by^38^

(Equation 20) $$S\left(\rho_{G}\right)=-\text{Tr}\left(\rho_{G}\;\ln\;\rho_{G}\right)=\sum_{j=1}^{2}s_{V}\left(\nu_{j}\right)\text{,}$$

with $\nu_{j},j=1,2\text{,}$ being the symplectic eigenvalues of the covariance matrix and

(Equation 21) $$s_{V}\left(x\right)=\left(x+\frac{1}{2}\right)\ln\left(x+\frac{1}{2}\right)-\left(x-\frac{1}{2}\right)\ln\left(x-\frac{1}{2}\right)\text{.}$$

For a two-mode Gaussian state the symplectic eigenvalues are given in terms of the symplectic invariants^38^ by:

(Equation 22) $$\nu_{\mp }^{2}=\frac{\Delta \mp \sqrt{\Delta^{2}-4\det\;\sigma }}{2}\text{,}$$

where $\Delta =\det\;\sigma_{a}+\det\;\sigma_{b}+2\det\;\sigma_{ab}$ is the seralian.

### Szilard engine performance

We describe now the dynamics of a bimodal Gaussian state in noisy channels. We work in the framework of the theory of open quantum systems, employing the Markovian Kossakowski-Lindblad master equation in the interaction picture for a state described by the density operator ρ (we set ℏ=1)^22^^,^^39^^,^^40^^,^^41^:

(Equation 23) $$\frac{d\rho }{dt}=\sum_{k=1}^{2}\frac{\lambda }{2}\left\{\left(N_{k}+1\right)L\left[{\hat{\xi }}_{k}\right]+N_{k}L\left[{\hat{\xi }}_{k}^{†}\right]-M_{k}^{*}D\left[{\hat{\xi }}_{k}\right]-M_{k}D\left[{\hat{\xi }}_{k}^{†}\right]\right\}\rho \text{,}$$

where ${\hat{\xi }}_{k=1,2}=\hat{a},\hat{b}$, ${\hat{\xi }}_{k}^{†}$ and ${\hat{\xi }}_{k}$ are the creation and the annihilation operators of the two bosonic modes. λ represents the damping parameter, and $N_{k}$ and $M_{k}$ are the effective photon numbers and, respectively, the squeezing parameters of the squeezed (phase-sensitive) environments. At thermal equilibrium $M_{k}=0$ and $N_{k}$ are the average numbers of thermal photons in the reservoirs. Lindblad superoperators are given by $L\left[\hat{O}\right]\rho =2\hat{O}\rho {\hat{O}}^{†}-\rho {\hat{O}}^{†}\hat{O}-{\hat{O}}^{†}\hat{O}\rho$ and $D\left[\hat{O}\right]\rho =2\hat{O}\rho \hat{O}-\hat{O}\hat{O}\rho -\rho \hat{O}\hat{O}$. The positivity of the density matrix imposes the constraints ${\left|M_{k}\right|}^{2}\leq N_{k}\left(N_{k}+1\right)$. A two-mode Gaussian state is completely characterized by its first and second-order moments:

(Equation 24) $$\begin{array}{cc} & \bar{X_{i}}=\left\langle {\hat{X}}_{i}\right\rangle \text{,}\\ & \sigma_{ij}=\frac{1}{2}\left\langle \left({\hat{X}}_{i}{\hat{X}}_{j}+{\hat{X}}_{j}{\hat{X}}_{i}\right)\right\rangle -\left\langle {\hat{X}}_{i}\right\rangle \left\langle {\hat{X}}_{j}\right\rangle ,i,j=1,\ldots ,4\text{.} \end{array}$$

The brackets ⟨…⟩ stand for the quantum average and $\hat{X}=\left(\hat{x_{a}},\hat{p_{a}},\hat{x_{b}},\hat{p_{b}}\right)$ denotes the vector of the canonical operators of the bipartite system. In the following we neglect the first order moments, since they can be made zero by suitable local displacements in the phase space. The evolution described by the master Equation 23 preserves the Gaussian character of the bimodal state. The time evolution of the covariance matrix of the considered state is given by^22^^,^^42^^,^^43^:

(Equation 25) $$\sigma \left(t\right)=e^{-\lambda t}\sigma \left(0\right)+\left(1-e^{-\lambda t}\right)\sigma \left(\infty \right)\text{,}$$

where σ(0) is the covariance matrix of the initial bimodal Gaussian state and σ(∞) is the asymptotic covariance matrix, which depends only on the environment parameters^44^:

(Equation 26) $$\sigma \left(\infty \right)=\underset{k=1,2}{⊕}\sigma_{k}\left(\infty \right)\text{,}$$

where

(Equation 27) $$\sigma_{k}\left(\infty \right)=\left(\begin{array}{cc} \left(\frac{1}{2}+N_{k}+M_{kR}\right)/\omega_{k} & M_{kI}\\ M_{kI} & \left(\frac{1}{2}+N_{k}-M_{kR}\right)\omega_{k} \end{array}\right)\text{.}$$

Here, $M_{kR}$ and $M_{kI},k=1,2\text{,}$ are the real and imaginary parts of $M_{k}$, respectively, with

(Equation 28) $$N_{k}=n_{th,k}\left(\cosh^{2}\;R+\sinh^{2}\;R\right)+\sinh^{2}\;R\text{,}$$

(Equation 29) $$M_{k}=-\left(2n_{th,k}+1\right)\cosh\;R\;\sinh\;R\;\exp\;i\varphi \text{,}$$

and $n_{th,k}=\frac{1}{2}\left(\coth\left(\frac{\omega_{k}}{2T}\right)-1\right)$ are the average numbers of thermal photons (we put Boltzmann constant $k_{B}=1$). For the two reservoirs, we consider the same temperature *T*, squeezing parameter *R* and squeezing phase φ. Our purpose is to estimate the performance of the Szilard engine based on an initial bimodal Gaussian state $\sigma \left(0\right)\equiv \sigma_{init}$ (10) evolving in Gaussian noisy channels, in the case when a squeezed thermal state (4) is used as the seed of measurement. We rewrite Equation 5 as follows^34^:

(Equation 30) $$\gamma^{\pi_{b}}=\frac{2N_{S}+1}{2}R\left(\zeta \right)S\left(\varrho \right)R^{T}\left(\zeta \right)\text{,}$$

where $R\left(\zeta \right)=\left(\begin{array}{cc} \cos\;\zeta & -\sin\;\zeta \\ \sin\;\zeta & \cos\;\zeta \end{array}\right)$ is the phase rotation matrix with ζ=θ/2 and $S\left(\varrho \right)=\left(\begin{array}{cc} \;\exp\;\left(-2\varrho \right) & 0\\ 0 & \;\exp\;\left(2\varrho \right) \end{array}\right)$ is the squeezing matrix. By introducing the strength of the measurement related to the squeezing of the seed of measurement μ=exp(−2ϱ), we obtain:

(Equation 31) $$\gamma^{\pi_{b}}=\frac{2N_{S}+1}{2}\left(\begin{array}{cc} \cos\;\zeta & -\sin\;\zeta \\ \sin\;\zeta & \cos\;\zeta \end{array}\right)\left(\begin{array}{cc} \mu & 0\\ 0 & \frac{1}{\mu } \end{array}\right)\left(\begin{array}{cc} \cos\;\zeta & \sin\;\zeta \\ -\sin\;\zeta & \cos\;\zeta \end{array}\right)\text{.}$$

μ=0 corresponds to a homodyne measurement and μ=1 corresponds to a heterodyne one. The expression of the extracted work as a function of the parameters characterizing the two-mode Gaussian state and the seed of the measurement is the following (for simplicity, we omit here to write the dependence on time *t*. Here, we use the standard form of a Gaussian state Equation 10:

(Equation 32) $$W\left(t\right)=\frac{T}{2}\ln\frac{a^{2}\left(1+2N_{S}+2b\mu \right)\left(2b+\mu +2N_{S}\mu \right)}{E}\text{,}$$

where

(Equation 33) $$\begin{array}{cc} E & =4c^{2}d^{2}\mu +a^{2}\left(1+2N_{S}+2b\mu \right)\left(2b+\mu +2N_{S}\mu \right)-a\left(c^{2}+d^{2}\right)\left(1+4b\mu +\mu^{2}+2N_{S}\left(1+\mu^{2}\right)\right)\\ & +a\left(c^{2}-d^{2}\right)\left(1+2N_{S}\right)\left(\mu^{2}-1\right)\cos\;\theta \text{.} \end{array}$$

In the case of the homodyne measurement (μ=0) this expression becomes

(Equation 34) $$\lim_{\mu \to 0}W\left(t\right)=\frac{T}{2}\ln\frac{2a\left(t\right)b\left(t\right)}{2a\left(t\right)b\left(t\right)-c^{2}\left(t\right)-d^{2}\left(t\right)+\left[-c^{2}\left(t\right)+d^{2}\left(t\right)\right]\cos\;\theta }\text{,}$$

In the case of the heterodyne measurement (μ=1), we obtain

(Equation 35) $$\lim_{\mu \to 1}W\left(t\right)=\frac{T}{2}\ln\frac{a^{2}\left(t\right){\left(1+2b\left(t\right)+2N_{S}\right)}^{2}}{\left(a\left(t\right)+2a\left(t\right)b\left(t\right)-2c^{2}\left(t\right)+2a\left(t\right)N_{S}\right)\left(a\left(t\right)+2a\left(t\right)b\left(t\right)-2d^{2}\left(t\right)+2a\left(t\right)N_{S}\right)}\text{,}$$

and in the limit of large values of the strength of the measurement (μ→∞) we get:

(Equation 36) $$\lim_{\mu \to \infty }W\left(t\right)=\frac{T}{2}\ln\frac{2a\left(t\right)b\left(t\right)}{2a\left(t\right)b\left(t\right)-c^{2}\left(t\right)-d^{2}\left(t\right)+\left[c^{2}\left(t\right)-d^{2}\left(t\right)\right]\cos\;\theta }\text{.}$$

We notice that in the case of homodyne detection the extracted work is independent of the number of thermal photons of the seed of the measurement, while in the case of heterodyne measurement the extracted work does not depend on the phase of the measurement. We observe also that, independently of the strength of the measurement, the extracted work does not depend on the phase of the measurement if c(t)=±d(t), i.e., if the two-mode Gaussian state preserves in time the form of a mode-mixed thermal state or a squeezed thermal state. In the limit of a large number of thermal photons of the seed of the measurement we easily obtain from Equation 32 that the extracted work tends to zero. Now let us consider the special case when at the initial moment of time the two-mode Gaussian state is a squeezed vacuum state ($n_{1}=n_{2}=0$), the modes are in resonance (for simplicity we set $\omega_{1}=\omega_{2}=1$) and the seed of measurement is a squeezed vacuum state ($N_{S}=0$). Then the extracted work is given by the simple expression

(Equation 37) $$W\left(0\right)=\frac{T}{2}{\text{lncosh}}^{2}2r\text{.}$$

**Figure 2:**
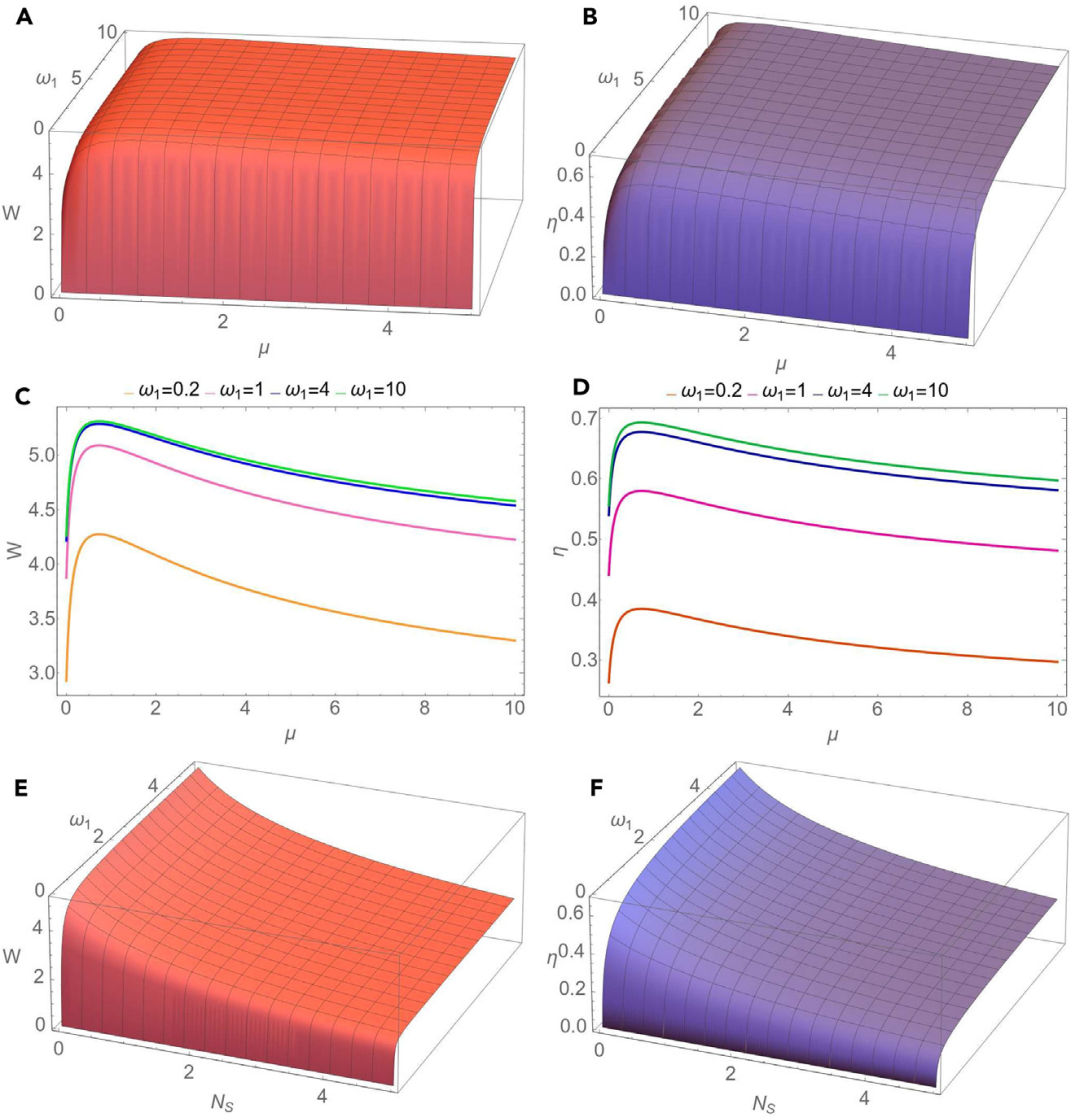
Dependence of the extractable work *W* (A) and information-work efficiency η. (B) on the strength of the measurement μ and the frequency of the first mode $\omega_{1}$ for a squeezed vacuum state ($N_{S}=0$) taken as the seed of measurement. Dependence of the extractable work. (C) and information-work efficiency (D) on μ for different values of the frequency $\omega_{1}$ and for $N_{S}=0$. Dependence of the extractable work (E) and information-work efficiency (F) on the number of thermal photons $N_{S}$ of the seed of measurement and the frequency $\omega_{1}$ for the strength of measurement μ=1. The other parameters are time t=0.5, squeezing between the modes r=1.8, thermal photon numbers of the modes $n_{1}=n_{2}=0$, environment temperature T=2, squeezing of the bath R=0.2, phase of the bath φ=π/4, frequency of the second mode $\omega_{2}=1$, phase of the seed of measurement θ=0 and dissipation parameter λ=0.1. The figure is obtained using Wolfram Mathematica 11.3.0.^45^

**Figure 3:**
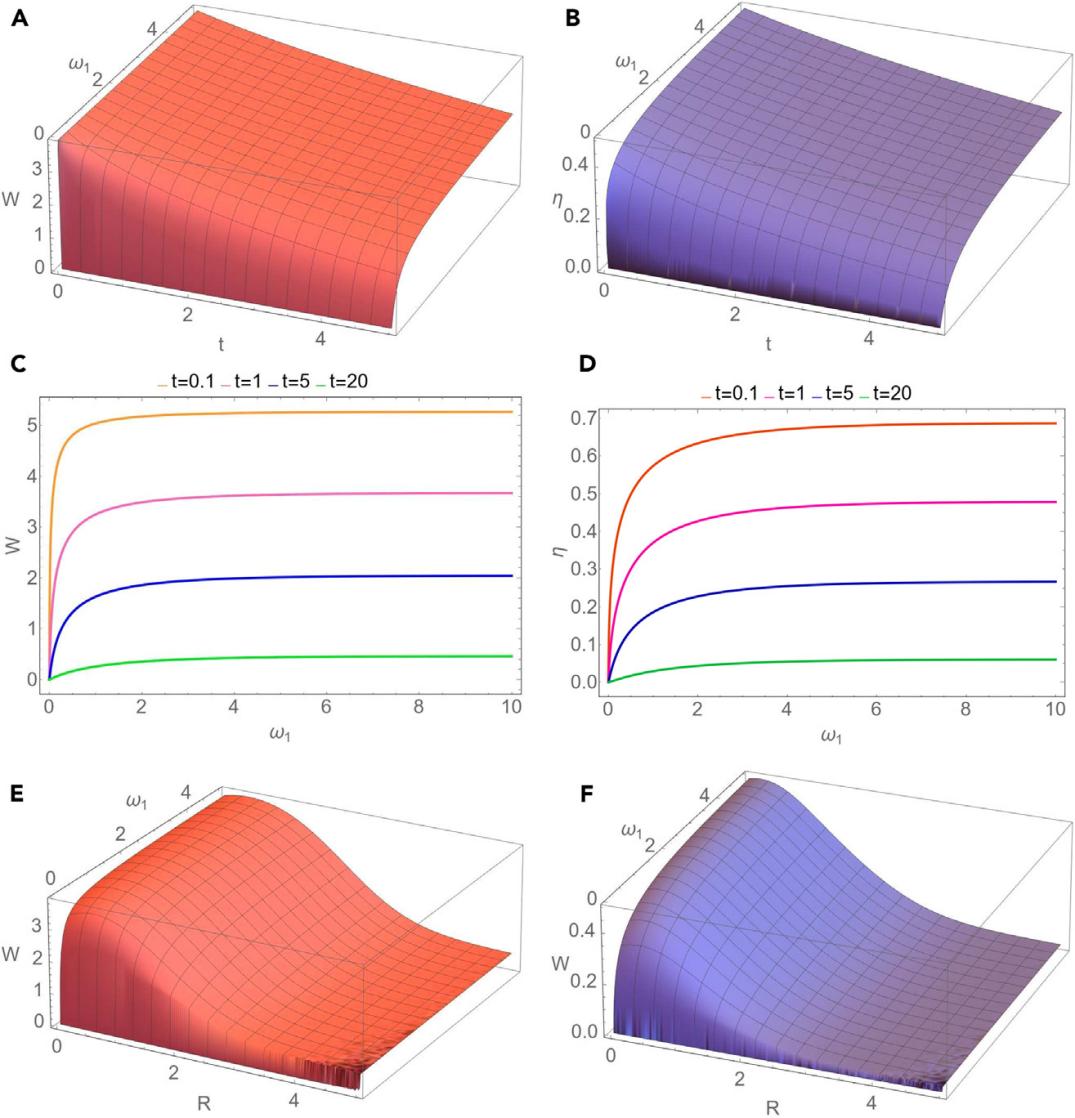
Dynamics of the extractable work *W* (A) and information-work efficiency η (B) with evolution time *t* and the frequency of the first mode $\omega_{1}$ for the squeezing of the noisy channel R=0.2, the number of average thermal photons of the seed of measurement $N_{S}=1$ and the strength of measurement μ=1. Dependence of the extracted work (C) and information-work efficiency (D) on the first mode frequency $\omega_{1}$ for different moments of time *t* ($N_{S}=0\text{,}\mu =0$). Dependence of the extracted work *W* (E) and information-work efficiency η (F) on the squeezing of the noisy channel *R* and the frequency of the first mode $\omega_{1}$ for time t=0.5, the number of average thermal photons of the seed of measurement $N_{S}=1$ and the strength of measurement μ=0.135. The other parameters are squeezing between the modes r=1.8, the number of thermal photons of the modes $n_{1}=n_{2}=0$, the environment temperature T=2, the phase of the bath φ=π/4, frequency of the second mode $\omega_{2}=1$, the phase of the seed of measurement θ=0 and dissipation parameter λ=0.1. The figure is obtained using Wolfram Mathematica 11.3.0.^45^

**Figure 4:**
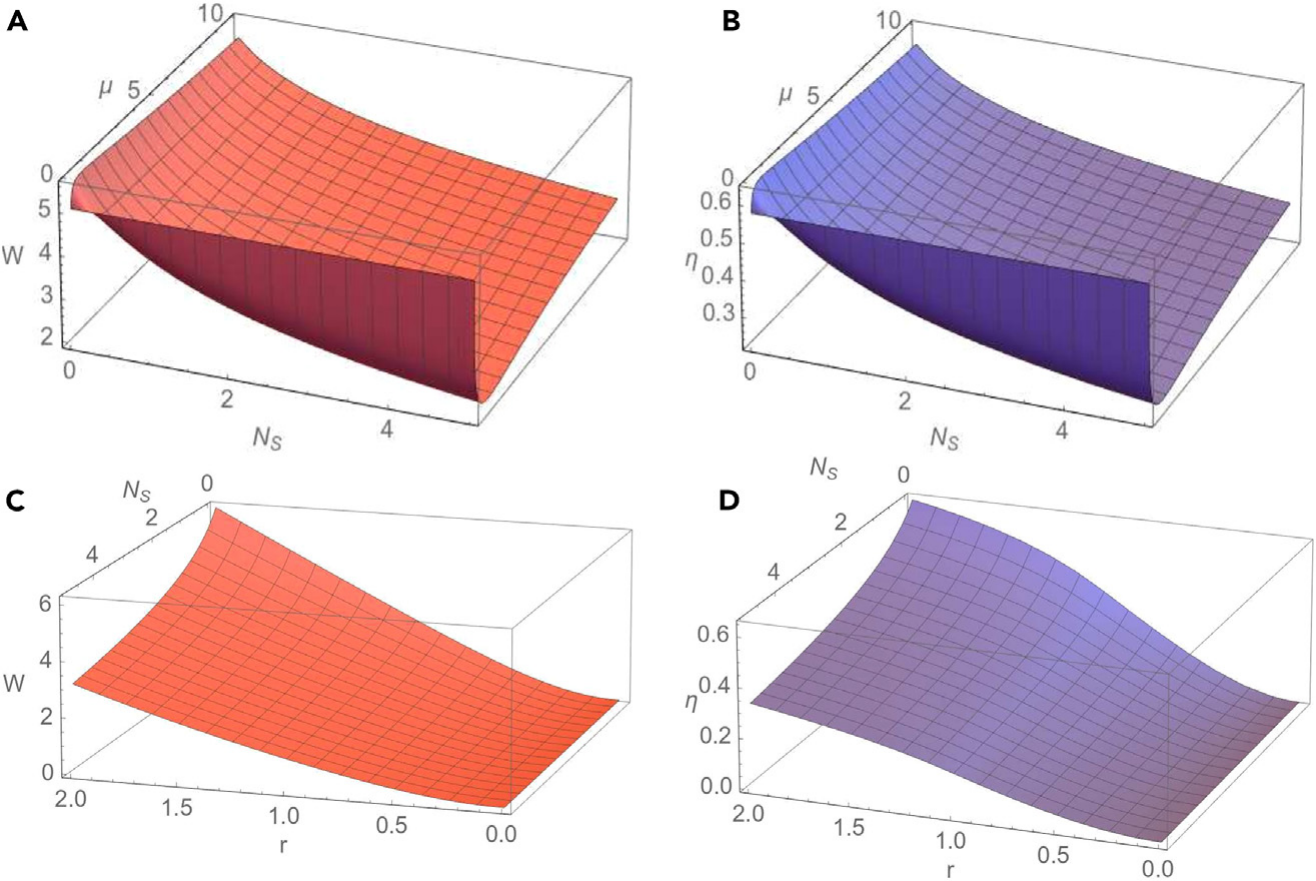
Dependence of the extractable work *W* (A) and information-work efficiency η (B) on the number of thermal photons of the seed of measurement $N_{S}$ and the strength of measurement μ for the squeezing between the modes r=1.8. Dependence of the extracted work *W* (C) and information-work efficiency η (D) on the squeezing between the two modes *r* and the number of thermal photons of the seed of measurement $N_{S}$ for the strength of measurement μ=0.135. The other parameters are time t=0.1, the number of thermal photons of the two modes $n_{1}=n_{2}=0$, frequencies of the two modes $\omega_{1}=\omega_{2}=1$, temperature of the bath T=2, squeezing of the bath R=0.2, the phase of the bath φ=π/4, the phase of the seed of measurement θ=0 and dissipation parameter λ=0.1. The figure is obtained using Wolfram Mathematica 11.3.0.^45^

**Figure 5:**
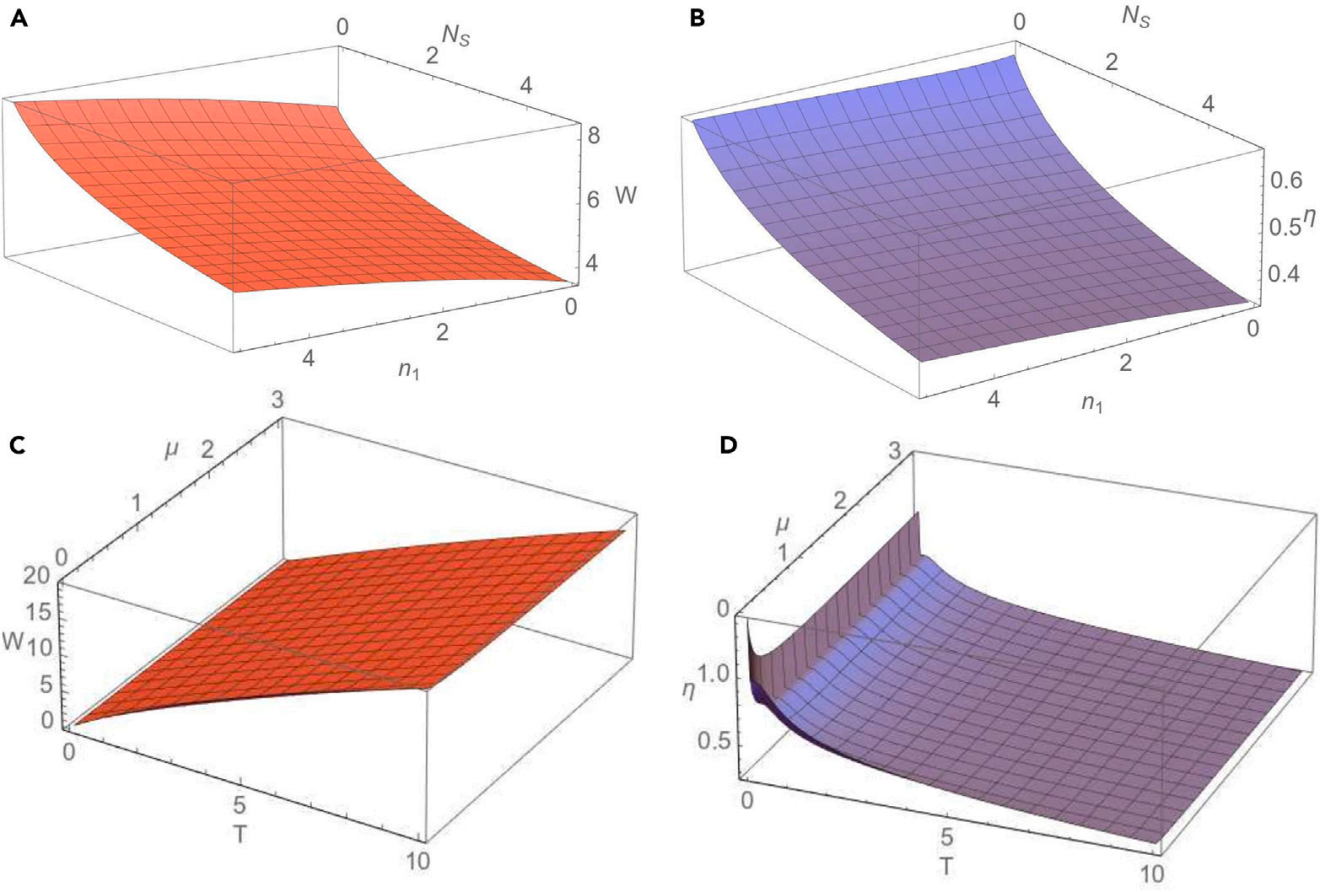
Dependence of the extractable work *W* (A) and information-work efficiency η (B) on the average number of thermal photons of the first mode $n_{1}$ and the average number of thermal photons of the seed of measurement $N_{S}$ for the average number of thermal photons of the second mode $n_{2}=1$, temperature of the bath T=2 and the strength of measurement μ=0.135. Dependence of the extracted work *W* (C) and information-work efficiency η (D) on the temperature *T* of the bath and the strength of measurement μ for the average number of thermal photons of the two modes $n_{1}=n_{2}=0$ and average number of thermal photons of the seed of measurement $N_{S}=1$. The other parameters are time t=0.1, squeezing between the modes r=1.8, number of thermal photons of the modes $n_{1}=n_{2}=0$, the squeezing parameter of the noisy channel R=0.2, the phase of the bath φ=π/4, frequency of the two modes $\omega_{1}=\omega_{2}=1$, phase of the seed of measurement θ=0 and dissipation parameter λ=0.1. The figure is obtained using Wolfram Mathematica 11.3.0.^45^

As will be seen in the obtained numerical results, for a given set of the parameters characterizing the initial Gaussian state, environment and the seed of measurement, the maximal value of the extracted work is reached just at the initial moment of time. Due to the interaction with the environment, the extracted work is decreasing during the time evolution. In addition, in the limit of large times the covariance matrix of the evolved system is given by Equation 26, therefore $\sigma_{ab}$ becomes zero matrix (c=d=0). Then it is easy to see from Equation 32 that the extracted work becomes zero. Using Equation 32, it can be shown that at the initial moment of time, in the case of a heterodyne measurement (μ=1), the extracted work reaches its minimal value for all values of the phase of the seed of measurement. In the Figure 2, we describe the behavior of the extracted work and information-work efficiency in dependence on the parameters characterizing the evolution of the two-mode Gaussian state in the squeezed thermal environment and the seed of measurement. In Figures 2A and 2B we illustrate the dependence of the extracted quantum work and information-work efficiency on the frequency of the first mode $\omega_{1}$ and the strength of measurement μ. For a definite moment of time, both extracted work and work efficiency manifest a similar behavior, namely they rapidly increase with frequency for relatively small values of $\omega_{1}$ and slowly for larger values of the frequency, till saturation. Likewise, for the considered values of the parameters, these quantities first rapidly increase with the strength of measurement for relatively small values of μ, and then they decrease by increasing the strength of measurement. The same behavior can be observed in Figures 2C and 2D), where it is represented the dependence of the extracted quantum work and information-work efficiency on the strength of measurement, for different values of the first mode frequency. In addition, in Figures 2E and 2F) it is shown the influence of the thermal photon number $N_{S}$ of the seed of measurement on the extracted quantum work and, respectively, on the efficiency. It is important to emphasize that the quantum work can be extracted even when the number of thermal photons of the seed of measurement is non-zero, so that the Szilard engine would function properly even in this situation. However, we notice that both the extracted work and information-work efficiency decrease by increasing the number of thermal photons and, as previously stated, the calculations confirm that they tend asymptotically to zero in the limit of large values of the thermal photon number. In addition to the previous description, we notice from Figures 3A and 3B that both the extracted work from the bimodal Szilard engine and the information-work efficiency decrease in time. In the limit of asymptotically large times, we see, by using Equations 27 and 32, that both these quantities tend to zero, independent of the values of the parameters characterizing the bipartite system, the environment and the seed of the measurement. In Figures 3C and 3D) we also present the dependence of the extracted work and, respectively, work-information efficiency on the frequency of the first mode at various moments of time. We notice that in the limit of large values of the frequency, both these quantities tend to a definite asymptotic value. The new information that is illustrated in Figures 3E and 3F), is that both extracted work and information-work efficiency decrease by increasing the squeezing parameter *R* of the squeezed thermal bath. Therefore, the squeezing of the environment acts toward the reduction of the extracted work and of its efficiency. In Figures 4 and 5 we consider the resonant case ($\omega_{1}=\omega_{2}=1$). The dependence of the extracted work using a bimodal Szilard engine as the working medium and of the information-work efficiency on the strength of measurement μ and the thermal number of photons $N_{S}$ of the seed of measurement is depicted in Figures 4A and 4B). One can see that for relatively small values of $N_{S}$ both these quantities increase with the strength of measurement μ for relatively small values of μ, while for larger values of μ they slowly decrease by increasing μ. Conversely, for relatively large values of $N_{S}$ both these quantities decrease by increasing the strength of measurement μ for relatively small values of μ, while for larger values of μ they increase with μ. In addition, in Figures 4C and 4D) we observe that both the extracted work and the information-work efficiency increase with the squeezing between the two modes. This behavior, determined by the squeezing between the modes is opposite to that determined by the squeezing of the environment, illustrated in Figures 3C and 3D. At the same time, we notice again that both the extracted work and the information-work efficiency decrease by increasing the thermal photon number $N_{S}$. In Figures 5A and 5B, we observe that both the extractable work and the information-work efficiency increase with the number of thermal photons $n_{1}$ of the first mode, for a given value of the thermal photon number $n_{2}$ of the second mode. Consequently, the bimodal squeezed thermal states are more suitable than the squeezed vacuum states to be used for the Szilard engine. On the other hand, the squeezed pure states for which $N_{S}=0$ are more convenient to use as the seed of measurement, since in this case one can obtain a better Szilard engine performance. This conclusion is also in agreement with that one already known in the literature.^32^ The extracted work increases with the temperature *T* of the environment, as one can see from Figure 5C), while, by contrary, from Figure 5D) we see that the information-work efficiency decreases by increasing the temperature. The explanation of this behavior consists in the fact that the erasure work increases much faster with the temperature than the extracted work. From these last plots, we can also see that, for the considered parameters, the strength of measurement does not essentially affect neither the extracted work nor the information-work efficiency.

### Conclusions

The main focus was the description of the influence of the seed of measurement, chosen as a squeezed thermal state, on the performance of a Szilard engine. We employed two entangled bosonic modes evolving in a Gaussian noisy channel as the engine working medium. We have investigated the behavior of the extracted quantum work and information-work efficiency of the Szilard engine as functions of the parameters of the working medium (frequencies, the average number of thermal photons and squeezing of the modes, temperature, squeezing and phase of the bath) and the POVM parameters (average number of thermal photons, strength and phase of the measurement). The analysis has been carried out based on the covariance matrix formalism. We used the Markovian Kossakowski-Lindblad master equation to study the evolution in time of the bimodal system in interaction with the squeezed thermal environment and Rényi entropy of order 2 to define extracted quantum work. A bimodal general Gaussian state was taken as the initial state of the system and a unimodal squeezed thermal state as the seed of measurement. The main results obtained in this paper can be summarized as follows. In the case of homodyne detection the extracted work is independent of the number of thermal photons of the seed of the measurement, while in the case of heterodyne measurement the extracted work does not depend on the phase of the measurement. If the two-mode Gaussian state preserves in time the form of a squeezed thermal state or a mode-mixed thermal state, then, independently of the strength of the measurement, the extracted work does not depend on the phase of the measurement. Both the extracted work and information-work efficiency decrease by increasing the number of thermal photons of the seed of measurement and they tend asymptotically to zero in the limit of large values of the thermal photon number. We have to mention that the extraction of quantum work is still possible in the presence of thermal noise in the seed of measurement, so that the Szilard engine would still function properly, however, better results can be obtained by using a squeezed pure state instead of a squeezed thermal state as a seed of measurement. For a given set of the parameters characterizing the initial Gaussian state, environment and the seed of measurement, the maximal value of the extracted work is reached just at the initial moment of time. Moreover, at the initial moment of time, in the case of a heterodyne measurement the extracted work reaches its minimal value, for all values of the strength of measurement and phase of the seed of the measurement. Due to the interaction with the environment, both the extracted work and the information-work efficiency are decreasing during the time evolution and in the limit of large times they become zero, for all values of the parameters characterizing the bipartite system, the environment, and the seed of the measurement. At a definite moment of time, both extracted work and work efficiency rapidly increase with the frequency of the modes for relatively small values and slowly for larger values of the frequency, till saturation. In general, due to the competition between the influences produced by the parameters characterizing the bimodal system, environment, and the seed of measurement, both extracted work and work efficiency manifest a non-monotonic behavior as functions of the strength of measurement. Both extracted work and information-work efficiency increase with the squeezing between the two modes and with the number of thermal photons of the modes. Consequently, the bimodal squeezed thermal states are more suitable than the bimodal squeezed vacuum states to be used for the Szilard engine. The extracted work increases with the temperature of the environment, while, by contrary, the information-work efficiency decreases by increasing the temperature. This behavior is due to the fact that the erasure work increases much faster with the temperature than the extracted work. Likewise, they decrease by increasing the squeezing parameter of the squeezed thermal bath, therefore, the squeezing of the environment acts toward the reduction of the extracted work and of its efficiency. In other words, we can say that the parameters describing the environment, namely temperature and squeezing of the bath, impair the engine performance.

## STAR★Methods

### Key resources table

**Table:** 

REAGENT or RESOURCE	SOURCE	IDENTIFIER
**Software and algorithms**		

Wolfram Mathematica 11.3.0	Wolfram Research, Inc.	https://www.wolfram.com/mathematica/
Wolfram Mathematica netbooks for numerical simulations.	Zubarev, A. (2023, September 15). Influence of the seed of measurement on the work extracted in a quantum Szilard engine. Retrieved from osf.io/sakb6	https://doi.org/10.17605/OSF.IO/SAKB6

### Resource availability

#### Lead contact

Further information and requests should be directed to the lead contact, Alexei Zubarev (alxzubarev@gmail.com).

#### Materials availability

The study did not involve any materials.

#### Data and code availability

• All data have been numerically generated.
• All calculations can be carried out using the equations presented in the main text.
• All softwares are listed in the key resources table.
• The code is available at osf.io/sakb6 (https://doi.org/10.17605/OSF.IO/SAKB6).

### Experimental model and study participant details

It is not applicable for this study.

### Method details

All methods are presented in the body of the article.

### Quantification and statistical analysis

It is not applicable for this study.
